# Homeostatic intrinsic plasticity, neural heterogeneity and memory maintenance

**DOI:** 10.1186/1471-2202-16-S1-P98

**Published:** 2015-12-18

**Authors:** Yann Sweeney, Jeanette Hellgren-Kotaleski, Matthias Hennig

**Affiliations:** 1 IANC, School of Informatics, University of Edinburgh, Edinburgh, UK; 2 Department of Computational Biology, KTH, Stockholm, Sweden

## 

Neural firing rates must be maintained within a stable range in the face of ongoing fluctuations in synaptic activity. This can be achieved through homeostatic intrinsic plasticity. However, here we show that such a mechanism, while successfully regulating neural firing rates, has an adverse effect on a network's ability to encode and retain memories. This is due to its interactions with Hebbian plasticity; neurons whose firing rates change following potentiation or depression of synaptic inputs will experience modifications in intrinsic excitability toward their homeostatic target, which can cause subsequent synaptic weight variations and disrupt learning. Essentially, this failure is a direct consequence of homeostasis preventing neural heterogeneity in order to maintain stable activity.

**Figure 1:**
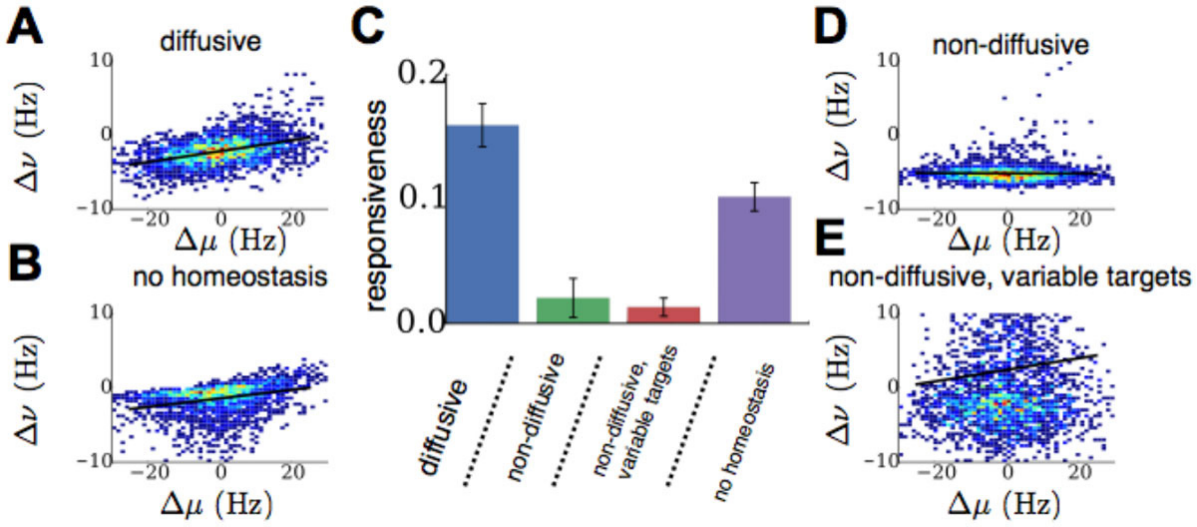
**Responsiveness of a network to changes in input when different types of homeostasis are used in order to reach a steady target state**. Panels A-B, D-E show firing rate changes Δν of all neurons in a network following input changes Δµ. Black lines show linear fit, with R^2 ^values used to quantify the average network responsiveness (panel C).

We propose a new mechanism, diffusive homeostasis, in which neural excitability is modulated by a diffuse messenger, specifically nitric oxide, which is known to freely cross cell membranes and homeostatically regulate neural excitability [1]. Information about a neuron's firing rate can be carried by nitric oxide, meaning that an individual neuron's excitability is affected by neighbouring neurons' firing rates as well as its own. We find that this allows a neuron to deviate from the target population activity, as its neighbours will counteract this deviation, thus maintaining stable average activity. We show that this form of neural heterogeneity endows a network with more flexibility than heterogeneity through variable target firing rates in individual neurons, which in turn leads to networks that are more responsiveness to changes in synaptic inputs (Figure 1) [2]. The increased flexibility in firing rates conferred by diffusive homeostasis resolves the conflict between homeostatic intrinsic plasticity and Hebbian plasticity by limiting the impact of homeostasis on individual synaptic modifications. Consequently, networks endowed with this diffusive mechanism have an improved learning capability compared to canonical, local homeostatic mechanisms, exhibit more stable synaptic weights, and allow for more efficient use of neural resources.

This work was supported by the Erasmus Mundus EuroSPIN programme (YS) and MRC Fellowship G0900425 (MHH).
